# Structural, Optical and Electrical Properties of Zinc Oxide Layers Produced by Pulsed Laser Deposition Method

**DOI:** 10.1186/s11671-017-2033-9

**Published:** 2017-04-04

**Authors:** G. Wisz, I. Virt, P. Sagan, P. Potera, R. Yavorskyi

**Affiliations:** 1 Rzeszow University, Rejtana 16C, 35-959 Rzeszow, Poland; 2 Drohobych State University, I. Franko, 24, 82100 Drohobych, Ukraine; 3 Vasyl Stefanyk PreCarpathian National University, T. Shevchenko, 57, 76018 Ivano-Frankivsk, Ukraine

**Keywords:** Thin film, Zinc oxide, Photoconduction of ZnO, Conductive thin films, Pulse laser deposition, 68.35-p, 68.37.Hk, 73.50.Pz

## Abstract

The structural, optical, and electrical properties of zinc oxide (ZnO) layers manufactured at different process conditions were investigated. ZnO epitaxial layers were grown on silicon, glass, and ITO/glass substrates by pulsed laser deposition (PLD) technique. The influence of power beam, substrate temperature, and deposition time on films properties was analysed. Morphological features of the film surface were investigated by scanning electron microscopy. A structural study shown planar orientation of films at low temperatures of substrate, but the columnar type of growth originated in temperature enhances. Electrical properties were determined in the temperature range 300–500 K. It was shown that the type of films conductivity is metallic and it is limited by charge transfer across grain boundaries.

## Background

Zinc oxide is one of the most important group II–VI semiconductor materials. It is a wide-bandgap oxide semiconductor with a direct energy gap of about 3.37 eV. ZnO has high chemical and mechanical stability; furthermore, it is nontoxic and widespread in nature. Recently, transparent-conducting oxides on the base of ZnO have been studied well [1–3]. ZnO is one of the most promising materials for the fabrication of the next generation of optoelectronic devices in the UV region and optical or display devices [4]. As a matter of fact, simultaneous occurrence of both high optical transmittances in the visible range and low resistivity make ZnO an important material for manufacturing of heat mirrors used in gas stoves, conducting coatings in aircraft glasses to avoid surface icing, and thin film electrodes in amorphous silicon solar cells. ZnO belongs to hexagonal wurtzite class; it is a semiconducting, piezoelectric, and optical waveguide material used in sensors, surface acoustic devices, transparent electrodes, and solar cells [5–7]. Controlling of ZnO physical properties depending on various factors, such as doping and temperature growth, is important for efficient function of devices on the base of ZnO structures. The existence of both (*n* and *p*) conduction types is of fundamental importance for application in light-emitting devices [8]. The nanostructures like nanotubes, nanorods, nanowalls, nanofibers and high-quality undoped and doped ZnO thin films have been grown with plasma-assisted molecular beam epitaxy, vapor transport deposition method, vacuum arc deposition metal organic chemical vapor deposition (MOCVD), sol–gel process, and spray pyrolysis [9, 10]. Such nanotubes, nanowires, nanoribbons, and nanofibers have deserved special attention for their potential applications in applied fields such as field emission displays, optical waveguides, solar cells, ultraviolet photodetectors, optical switches, and gas sensing [1–8]. The chemical bath deposition and sol–gel technique are also well known methods of preparation of ZnO thin films. Among these methods, spray pyrolysis is useful in wide range of applications [11, 12]. This method is cheaper, simpler and permits to obtain films for optoelectronic applications with required properties. Structural, electrical, and optical properties dependence on thickness of ZnO films has been investigated. The unique and fascinating properties of nanostructured materials have triggered tremendous motivation among scientists to explore the possibilities of using them in technological applications. In particular, the electronic and optical properties of nanostructure materials have been of great interest because of their potential applications in the fabrication of microelectronic and optoelectronic devices [13].

In this paper, the electrical, structural, and optical properties of ZnO nanostructured thin film deposited by PLD method and their changes during annealing have been investigated.

## Methods

The ZnO films grown on silicon, glass, and ITO/glass were deposited by the PLD method. The YAG: Nd^3+^ laser with the 532 nm (II harmonics) wavelength, 6 ns pulse time, and 16 J/cm^2^ fluence was used. The laser beam was focused on the target using a quartz lens with focal distance of 600 mm. The pressed ZnO powder was used as a target. The growth temperature *T*_s_ was kept in the range 20–400 °C and the deposition of the layers was carried out at 10^−8^ mbar vacuum.

Structural properties and cross-sectional images of the film were investigated by scanning electron microscopy Vega3 Tescan, for samples growth at the temperature *T*_s_ = 200 and 300 °C. The electrical characterization was carried out in a four probe conductivity cells. A constant voltage was applied to the sample and the current was measured using a Keithley electrometer. Current dependences on temperature were recorded during the cooling as well. When the samples reached the room temperature, it was taken to second heating–cooling cycle. During the second heating–cooling cycle, also the current dependence on temperature was recorded. The cycle curves were measured for films as grown and after annealing in oxygen atmosphere in 250 °C. Optical transmission spectra were measured in the wavelength range of 190–1100 nm using UNICAM UV300 spectrometer. The photoconductivity of thin films ZnO was determined by measuring resistance/conductivity of the films using the illumination by light-emitted diode with 365 nm.

## Results

### Structural Properties

**Fig. 1:**
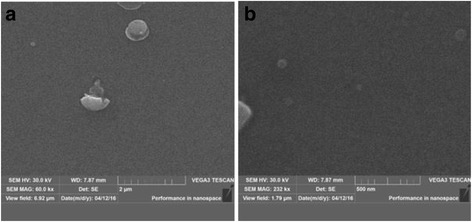
The surface morphology of ZnO/silicon samples deposited at the different substate temperatures *T*_s_: **a** 200 °C; **b** 300 °C

**Fig. 2:**
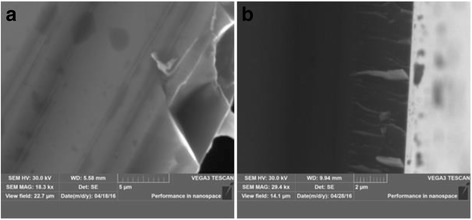
The cross-sectional images of ZnO films deposited at the different substrate temperatures *T*_s_: **a** 200 °C; **b** 300 °C

Surface morphology and cross section of ZnO samples deposited at growth temperature *T*_s_ = 200 and 300 °C are presented in Figs. 1 and 2, respectively. There is spherical precipitate with 680 nm size on the thin films surface which shows the uniform structure. At higher growth temperature, the structure of ZnO films is more uniform (Fig. 1b) and the size of spherical precipitate at *T*_s_ = 300 °C is less than at *T*_s_ = 200 °C.

Formation of the layer structure is observed at *T*_s_ = 200 °C on the cross section of ZnO thin film (Fig. 2a). At higher substrate temperature *T*_s_ = 300 °C, the columnar type of growth is observed for ZnO films (Fig. 2b). Columnar growth of the thin films indicates that ZnO films have *c*-axis preferred orientation.

### Electrical Characterization

The photoresponse of ZnO consists of two parts: a rapid process of photogeneration and recombination of electron-hole pairs and a slow process attributed to the oxygen adsorption and photodesorption on the film surface as well as the grain boundaries.

**Fig. 3:**
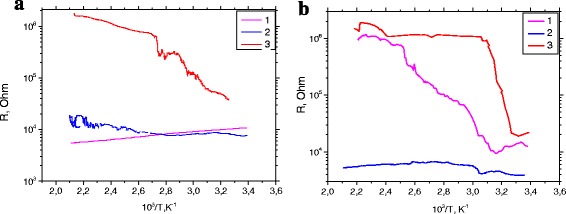
Change in resistance of the samples (*1* PLD, *2* PLD-200A, and *3* PLD-300) heating for as-growth thin films (**a**) and annealed (**b**)

Columnar growth with temperature increasing is accompanied by increase of the layer resistance value which was observed on the plots *R* (*T*) for sample at the *T*_s_ = 300 °C (Fig. 3a). Increase of average distance between the crystal columns affects increasing potential barrier at the grain boundaries and thus increasing resistance of the layer in the plane of the substrate. For the layer planar structure at *T*_s_ = 200 °C, increasing of resistance is not so clearly observed.

For the sample obtained at *T*_s_ = 300 °C after annealing (Fig. 3b), the clear and sharp increase of cross-resistance layer with the increase of temperature is observed. This behavior can be caused by a large number of clear boundaries between the crystalline ZnO columns. On the other hand, for a sample obtained at *T*_s_ = 200 °C (Fig. 3b), the decrease of resistance with increasing temperature which may indicate the different orientation of crystallite is obtained.

The resistivity value of 0.95 × 10^−1^ Ωcm (polycrystalline thin films obtained at *T*_s_ = 300 °C), is about one order higher than the resistivity of films obtained by other methods, including high-frequency sputtering oxide target. Semiconductor behavior has films grown only at low temperatures. The presence of metal conductivity in films and blue shift of the optical bandgap indicate the presence of relatively high carrier concentration. This can be caused by annealing and the effective concomitant decrease of the oxygen defects in ZnO. As-grown and annealed films show different scattering mechanisms. There are some types of oxygen defects in these films, such as oxygen defects, oxygen vacancies (*V*_O_), and interstitial oxygen atoms (*O*_i_). Annealing in oxygen atmosphere allows the reduction of a number of point defects. Metal conductivity determines the behavior of carrier scattering in severely defective degenerate semiconductors, while the semiconductor character defines the activation processes. In general, the degenerate of electronic conduction in ZnO thin films is a combination of both processes: scattering of carriers and their activation. Thus, structural properties of thin films of ZnO can be determined from the temperature dependence of electrical resistance as shown in Fig. 3. In this case, the value of the carrier concentration *n*_0_ is supposed to be dependent weakly on temperature and it caused by intrinsic defects such as oxygen vacancies and interstitial zinc. In these films, the mobility depends initially on the temperature, increases to a maximum, and then decreases with increasing temperature [14].

In the presence of various scattering mechanisms that control the mobility of electrons in ZnO polycrystalline thin films, mostly limited mobilities are scattering grain (grain) *μ*_*g*_, scattering lattice *μ*_*l*_, and (at low temperatures) ionized impurity scattering *μ*_*ii*_. Thus, the total mobility is given by

1 $$ \frac{1}{\mu}=\frac{1}{\mu_l}+\frac{1}{\mu_g}+\frac{1}{\mu_{ii}} $$

At higher temperatures, which are used in these studies, a significant contribution to the mobility makes the lattice scattering, as can be expected. It is determined by the expression:

2 $$ {\mu}_1={\left(\frac{\pi}{3}\right)}^{1/3}\cdot \frac{e{ h}^3{C}_l}{{\left({m}^{*}\right)}^2{E}_d{}^2 k{}_B T}\cdot \frac{1}{n^{1/3}} $$

Here, *h* is Planck’s constant, *k* is Boltzmann’s constant, m* is the effective mass of electron, *T* is absolute temperature, *E*_*d*_ is deformation potential constant, *C*_*l*_ is the elasticity modulus of system, and *n* is the concentration of electrons. Thus, the equation can be written in a short form ${\mu}_1=\frac{B}{T}\cdot \frac{1}{n^{1/3}}$ (*B* is some constant) that determines the mobility grid proportional to the reciprocal temperature. The experimental curves show linear fit conductivity of *T* ^−1^, and it confirms the superiority of lattice scattering in the range of high temperatures of 300 to 400 K. In addition, the slope changing of straight line is consistent with polycrystalline films grown at higher temperatures. Another scattering temperature mechanism mode operates at room temperature (300 K), which limits the electron mobility due to grain boundaries scattering (GBS). It is assumed that this is due to the column growth form at high substrate temperatures and increasing role of grain boundaries in the film annealing in an oxygen atmosphere.

The correlation between the structural characteristics, properties, and photovoltaic electric transport properties of films were evaluated by measuring of photoresponse of films, especially under UV irradiation.

**Fig. 4:**
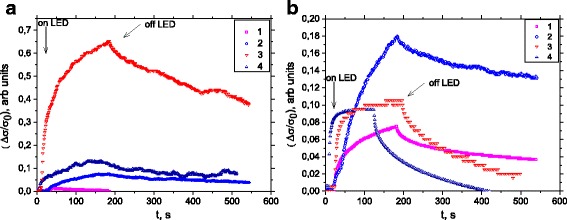
Photoconduction of the samples (*1* PLD, *2* PLD-200A, *3* PLD-300, and *4* PLD-400) thin film ZnO as-growth (**a**) and annealed (**b**)

The photoconductivity response was performed with the illuminated light of 365 nm. The curves of photoconductivity response of a ZnO films grown at temperature *T*_s_ = 20–400 °C is shown in Fig. 4a. The photocurrent for thin films with *T*_s_ = 300 °C rises within 3 min and falls to 50% of its maximum value within 6 min.

A very pronounced photoconductivity effect is observed in Fig. 4b for samples *T*_s_ = 300 and 400 °C after the annealing. There is an extremely rapid excitation and fast relaxation occurring during the 280 s for the sample *T*_s_ = 400 °C.

### Optical Properties

**Fig. 5:**
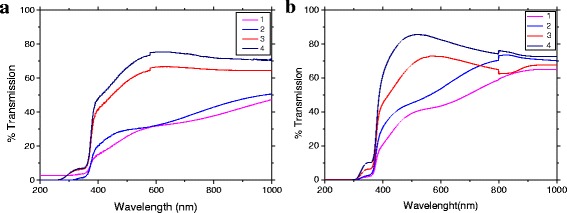
Optical transmission of the samples (*1* PLD, *2* PLD-200A, *3* PLD-300, and *4* PLD-400) thin film ZnO as-growth (**a**) and annealed (**b**)

Optical characterization of thin films gives information about other physical properties, e.g., bandgap energy, band structure, and optically active defects. The effects of thickness and annealing on the optical transmittance and the bandgap (*E*_*g*_) values of the ZnO films have been studied. The optical transmission (*T*, %) of the ZnO films formed at the growth temperature *T*_s_ = 20–300 °C is shown in Fig. 5a and after anneals in Fig. 5b. The transmission decreases sharply in the near ultraviolet region due to the bandgap absorption. Absorption edge takes place around 350 nm for all samples. Note that these films exhibit transparency in the visible range of the average transmittance, which lies between 30 and 70%. In addition, the lack of interference fringes in the transmission spectra is due to surface roughness, tower height, and scattering at the grain boundaries. Also, there is the skip increases at low-temperature annealing of films. Increased optical transmission associates with a decrease in oxygen defects. In parallel, the absorption tails can be observed in the visible region, which is characteristic for disordered systems (e.g., glassy) [15].

The samples have an average optical transparency of 60–70% in the region from 800 to 1100 nm and a sharp edge adsorption after annealing (Fig. 5b), which for all samples is approximately 360 nm. In order to obtain the bandgap, the absorption coefficient was calculated from the transmission data using the following relation:

3 $$ \alpha =\frac{ \ln \left(1/ Tr\right)}{d} $$

where *d* is the film thickness and *Tr* is the transmittance. On the other hand, beyond the band edge, the absorbance is very small and the transmittance is high. This indicates a low amount of impurities and a few lattice defects in obtained films. Also, the flat range of the transmission curves without interference fringes emphasizes the surface uniformity with small crystallite size. Here, the fabricated ZnO films are considered as a material having direct bandgap energy [16]. For the direct transition, the optical bandgap energy of ZnO film was determined using the equation:

4 $$ {\left(\alpha h\nu \right)}^2= A\left( h\nu -{E}_g\right) $$

where *A* is a constant, and *hν* is the photon energy, and *E*_*g*_ is the optical energy gap.

**Fig. 6:**
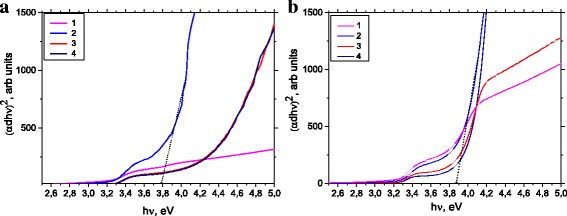
Optical energy gap of the samples (*1* PLD, *2* PLD-200A, *3* PLD-300, and *4* PLD-400) thin films ZnO as-growth (**a**) and annealed (**b**)

Figure 6 shows a graph (*αhν*)^2^ versus photon energy, allowing assessing the value of the energy gap with a sharp edge absorption using linear approximation. Estimated values of optical energy gap *E*_*g*_ of ZnO thin films are shown in Table 1. The results show an increase in the optical energy gap with increase of deposition temperature. This increase in the energy gap can be according to the considerable shift Burstein electron density [17]. Presence of tail absorption profile in the visible range gives Urbach energy *E*_*u*_, induced optical absorption on own defects. It can be estimated from the empirical Urbach law [18],

**Table 1:** The variation of bandgap energy and Urbach’s energy of ZnO thin films

Sample	*T*_s_, °C	*E*_*g*_, eV	*E*_*u*_, eV	Sample (anneal)	*E*_*g*_, eV	*E*_*u*_, eV
PLD	20	3.30	0.14	PLD an	3.58	0.27
PLD-200	200	3.79	0.36	PLD-200 an	3.87	0.32
PLD-300	300	4.08	0.18	PLD-300 an	3.83	0.12
PLD-400	400	4.19	0.28	PLD-400 an	3.98	0.28

5 $$ \left\{\begin{array}{l}\alpha \left( h\nu \right)={\alpha}_0{e}^{\frac{hv}{E_U}}\\ {}{E}_U=\alpha \left( h\nu \right){\left[\frac{d\left[\alpha \left( h\nu \right)\right]}{d\left[ h\nu \right]}\right]}^{-1}= h{\left[\frac{d}{d v}\left( \ln \alpha \left( h\nu \right)\right)\right]}^{-1}\end{array}\right., $$

where *α* represents the experimentally determined optical absorption profile and *α*_0_ is constant. *E*_*u*_ parameter is defined as the reciprocal value of the recession spectral characteristics for different films presented in Table 1. That formation of tail states zone in the forbidden zone indicates the spin-exchange interaction between the conduction electrons and electrons localized at defects and their interaction with phonons.

## Discussion

As described above, the surface conduction mechanism is mainly related to the absorption or desorption of chemisorbed oxygen. In the dark state, chemisorbed oxygen molecules capture free electrons: O_2_(g) + *e* ^−^ → O_2_^−^. This process leads to the formation of the depleted surface layer and causes the bending energy as areas of conduction and valence band. Formation of large amounts of ionized oxygen on the surface of ZnO increases the bending zones, leading to the formation of a potential barrier, and therefore to increased resistance. In terms of lighting UV light photos generated and adsorbed (O_2_) particles released: O_2_(g) + *hν* → O_2_^−^ + *e* ^−^. O_2_ neutral molecules embedded in the grain boundaries form oxygen ions and free electrons in the processes associated with polycrystalline. In this case, released by the light, the generated electrons contribute to the photoconductivity. The processes related to the intergrain boundaries are responsible for the faster process than the surface processes because of the large number of structural defects and defects in grains. In particular, the ZnO film, which is characterized by very small grains, can save a significant amount of adsorbed oxygen at the grain boundaries, which leads to higher number of carriers. In contrast, samples obtained at higher temperatures have the biggest grains; therefore, the number of carriers is decreased with trap number as expected with a fast response time. Thus, the current recession should be described by two mechanisms. The first one is related to the process of electron-hole recombination by chemisorbed oxygen atoms and a damping mechanism that is essentially independent of grain size and also depends on the temperature. The second one decay occurs through a process of recombination of electron-hole pairs by chemisorbed oxygen and defects on the surface. This process is generally much slower than the previous one. The decay time increases with temperature of ZnO deposition. This can be explained by taking into account the amount of chemisorbed oxygen on the surface at a higher temperature deposition. Thus, the grain size and the amount of adsorbed oxygen are probably the main parameters, which mainly manage the properties of photoresponse decay.

## Conclusions

ZnO thin films have been successfully deposited using pulsed laser deposition technique at different substrate temperatures that vary in the range of 20–400 °C. The experimental curves show a linear dependence of the electrical conductivity versus reciprocal temperature, and this confirms the superiority scattering lattice. This is the columnar film growth at higher substrate temperatures. At room temperature, electron mobility is limited by scattering grain boundaries. Photoconductivity curves are characterized by two distinct trends in photocurrent decay with a time constant that depends on the mechanism of recombination of nonequilibrium carriers.

## Abbreviations

ITO: Indium tin oxide
MOCVD: Metal organic chemical vapor deposition
PLD: Pulsed laser deposition
